# Current sensorless position-tracking control with angular acceleration error observers for hybrid-type stepping motors

**DOI:** 10.1038/s41598-022-19250-0

**Published:** 2022-09-02

**Authors:** Seok-Kyoon Kim, Kwan Soo Kim, Dong Kyu Lee, Choon Ki Ahn

**Affiliations:** 1 Department of Creative Convergence Engineering, Hanbat National University, Daejeon, 341-58 Korea; 2 School of Electrical Engineering, Korea University, Seoul, 136-701 Korea

**Keywords:** Mechanical engineering, Electrical and electronic engineering

## Abstract

This paper exhibits an advanced observer-based position-tracking controller for hybrid-type stepping motors with consideration of parameter and load uncertainties. As the main contribution, a current sensorless observer-based pole-zero cancellation speed controller is devised for the outer loop position-tracking controller including the convergence rate boosting mechanism. The features of this study are summarized as follows; first, the pole-zero cancellation angular acceleration error observer for the inner loop speed controller, second, the pole-zero cancellation speed control forcing the order of the controlled speed error dynamics to be 1, and, third, the outer loop position control incorporating the first-order target tracking system with its convergence rate booster. The resultant effectiveness is verified on a 10-W stepping motor control system.

## Introduction

The major advantages of stepping motors are the elimination of brushes and the use of a simple position regulation method to count the pulse numbers. These allow various industrial position control applications, such as computerized numerical control (CNC) machines, nuclear reactor control rods, robot arms, and printers^1–7^.

Implementing position and speed regulation with stepping motors is possible without any feedback sensors by counting the pulse numbers and adjusting the pulse frequency; however, their precision is predominantly reliant on the teeth numbers. At high-speeds, a stepping motor can experience mechanical problems such as step-out, resonance, and reversal of speed^8^. To overcome these, a micro-stepping technique with a partial closed-loop structure was proposed that determines the voltage update law statically while assuming the current-loop transfer function as 1. The corresponding closed-loop control precision and performance are dependent on the current controller. A proportional-integral-derivative (PID) control constitutes the current-loop for each phase with a well-tuned feedback gain using Bode and Nyquist techniques^8–12^. To maintain the desired performance across a large operation range, the resultant feedback gain must be magnified by increasing the motor speed, which is proportional to the back-electromotive force (EMF) disturbance. Parameter-dependent feed-forward compensators deal with this problem by canceling the motor-speed-dependent disturbance, which can achieve significant performance improvement in the high-speed mode^13^. A novel current-control technique was proposed based on the incorporation of a disturbance observer (DOB) in the sliding-mode control (SMC) to improve the feed-forward terms by reducing the parameter dependence; the proof of closed-loop convergence was presented by the Lyapunov stability theorem^14^. Another recent study established the elimination of current feedback sensors by combining feedback-linearization (FL) control and a passive observer driven by the position error, which included closed-loop stability analysis^15^. The level to which parameters depend on these techniques can be lowered by using the novel online parameter identifiers as in^16–21^. Interestingly, the position dynamics were considered, which transformed the entire machine dynamics into a linear-time varying system that could be stabilized by an $H_2$ controller with a passive observer^22^.

Unlike the aforementioned approaches (designed in the *a*-*b* axis), the introduction of a rotational *d*-*q* transformation simplifies the controller design task considerably by removing of the nonlinearities of the model that rely on the motor position^23^. This method also enables to enlarge the operation range by controlling the negative *d*-axis current^24^. Moreover, several recent techniques for three-phase permanent machines, as in^25–30^, can be considered as stabilizing solutions. In FL methods^31,32^, the position regulation task was transformed into a third-order nonlinear dynamics stabilization problem that required inverse dynamics with perfect machine parameter knowledge. Parameter updating mechanisms have been incorporated into an FL controller to reduce the regulation errors with the closed-loop system order increment^33^. The energy-shaping approach combining the two techniques of flatness and passivity alleviates the dependence on inverse dynamics and parameters, using the function of open-loop energy^34^. Sliding mode control (SMC) that forces closed-loop dynamics into the desired surface with the suppression of disturbances from model-plant mismatches is available; here, a discontinuous function with a conservative gain is used in the feedback loop^35–37^. Closed-loop performance improvement can be achieved by incorporating a learning part for the feedback-loop in the back-stepping controller that minimizes the cost-function using a learning algorithm^38^. Another learning control was suggested with compensation for the *q*-axis current reference using a repetitive space-learning technique. The tracking performance improvement for sinusoidal references was only observed from numerical simulations^39^. The recent DOB-based proportional-type positioning technique tried to robustly provide boosting to the closed-loop cut-off frequency in its transient periods, which requires current feedback and could limit the closed-loop performance limitation due to the absence of integral actions^40,41^.

From this literature survey, the accurate machine model information and current feedback remain as the practical problems to be handled in this study. The machine parameters are decomposed as their nominal and variations to lower the system model dependence for controller design task. The acceleration error observer removes the requirement of the current feedback loop without any machine model information. The contributions of this study are as follows:

- The proposed observer estimates the angular acceleration error by adopting the specially structured observer gain to invoke the first-order pole-zero cancellation for the estimation error dynamics, independent from the machine model information,
- The inner loop controller robustly stabilizes the speed and estimated acceleration errors in accordance the first-order dynamics, involving the order reduction property obtained from the pole-zero cancellation through the active damping compensation, and
- The outer loop adopts the convergence rate booster to reinforce the position-tracking performance by increasing the target system feedback gain proportional to the tracking error.

The prototype control system including a commercial 10-W hybrid-type stepping motor validates the effectiveness of the proposed technique in various scenarios.

## Hybrid-type stepping motor model

The stator of the stepping motors includes *a*- and *b*-phase, whose phase current and voltage are denoted as $i_x$ and $v_x$, $x=a,b$, respectively. Applying the orthogonal coordinate transformation with the rotor position $\theta$ and each phase teeth number $N_r$, respectively, it holds that^9,10,42^

1 $${\dot{\theta }}= \omega ,$$

2 $$J{\dot{\omega }}= - B \omega + T_e(i_q) - T_L,$$

3 $$L{\dot{i}}_x= - R i_x + p_x (i_d,i_q,\omega ) + v_x,~x=d,q,$$

$\forall t \ge 0$, with the state variables : $\theta$ - rotor position (rad), $\omega$ - rotor speed (rad/s), and $i_x$, $x=d,q$ - current (A) and control variable : $v_x$, $x=d,q$ - voltage (V). The output torque $T_e (i_q)$ (Nm) is proportional to the *q*-axis current as $T_e(i_q) \,{:=}\, K_m i_q$ with the torque coefficient $K_m$. The load torque $T_L$ (Nm) acts as the mismatched disturbance depending on the operation conditions. The disturbances $p_x (i_d,i_q,\omega )$ in modeling the back-EMF effect are defined as $p_d (i_d, i_q, \omega ) \,{:=}\, L N_r \omega i_q$ and $p_q (i_d, i_q, \omega ) \,{:=}\, -(L N_r i_d + K_m)\omega$ with the stator inductance *L* (H). The remaining machine parameters are given by: *J* - inertia moment of the rotor (kgm$^2$), *B* - viscous friction (Nm/rad/s), and *R* - stator resistance ($\Omega$).

To deal with the variations of parameter and load torque, nominal parameters denoted as $(\cdot )_0$ are introduced for the speed and current dynamics (2)–(3) to be expressed as

4 $$\begin{aligned} J_0 {\dot{\omega }}= - B_0 \omega + T_{e,0}(i_q) + {\bar{d}}_\omega \end{aligned}$$

5 $$\begin{aligned} L_0 {\dot{i}}_x= - R_0 i_x + p_{x,0} (i_d, i_q, \omega ) + v_x + {\bar{d}}_x, \end{aligned}$$

$x=d,q$, with $T_{e,0}(i_q) \,{:=}\, T_e(i_q)|_{K_m = K_{m,0}}$, $p_{x,0}(i_d,i_q,\omega ) \,{:=}\, p_{x}(i_d,i_q,\omega )|_{L= L_0, K_m = K_{m,0}}$, and lumped disturbances ${\bar{d}}_\omega$, ${\bar{d}}_d$, and ${\bar{d}}_q$. The following section presents the development of the position regulation law with the dynamics (4) and (5).

## Position-tracking control law

This study adopts the target position tracking behavior denoting $\theta ^*$ (different from the actual position measurement $\theta$) as the first-order system given by

6 $$\begin{aligned} \dot{\tilde{\theta }}^* = -\omega _{pc} \tilde{\theta }^*,~\forall t \ge 0, \end{aligned}$$

for the error $\tilde{\theta }^* \,{:=}\, \theta _{ref}-\theta ^*$, reference trajectory $\theta _{ref}$, and convergence rate $\omega _{pc} > 0$ (constant). The system (6) accomplishes the tracking objective; that is $\lim _{t\rightarrow \infty }\theta ^* = \theta _{ref}$, exponentially for any reference trajectory $\theta _{ref}$ according to the convergence specification $\omega _{pc}$. Therefore, the tracking controller is designed to guarantee the control objective: $\lim _{t\rightarrow \infty } \theta = \theta ^*$, exponentially, which is proved by analyzing the closed-loop dynamics in “Analysis” section.

### Outer loop

#### Convergence rate boosting mechanism

The time -varying convergence ratio ${\hat{\omega }}_{pc}$ replaces its constant version $\omega _{pc}$ in (6) as

7 $$\begin{aligned} \dot{\tilde{\theta }}^*= - {\hat{\omega }}_{pc} \tilde{\theta }^*, \end{aligned}$$

8 $$\begin{aligned} \dot{{\hat{\omega }}}_{pc}= \gamma _{pc}( (\tilde{\theta }^*)^2 + \rho _{pc}\tilde{\omega }_{pc} ),~\forall t \ge 0, \end{aligned}$$

where ${\hat{\omega }}_{pc}(0) = \omega _{pc}$, $\tilde{\omega }_{pc} \,{:=}\, \omega _{pc} - {\hat{\omega }}_{pc}$, and two design parameters $\gamma _{pc} > 0$ and $\rho _{pc} > 0$ determine the convergence rate booting and restoring natures, respectively. The time-varying nature of ${\hat{\omega }}_{pc}$ by the rule (8) makes the stability issue questionable, which is addressed in “Analysis” section with the boundedness property ${\hat{\omega }}_{pc} \ge \omega _{pc}$, $\forall t \ge 0$.

#### Position control

The manipulation $\omega = \omega _{ref} - \tilde{\omega }$ with $\tilde{\omega } \,{:=}\, \omega _{ref} - \omega$ and error $\tilde{\theta } \,{:=}\, \theta ^* - \theta$ give the error dynamics as $\dot{\tilde{\theta }} = - \omega _{ref} + \tilde{\omega } + {\dot{\theta }}^*$, $\forall t \ge 0$, whose stabilization can be established by the proportional-type stabilizing law:

9 $$\begin{aligned} \omega _{ref} = \lambda _{pc} \tilde{\theta } + {\dot{\theta }}^*,~\forall t \ge 0, \end{aligned}$$

with the adjustable convergence rate $\lambda _{pc} > 0$. Note that the compensation term ${\dot{\theta }}^*$ is obtainable from the implementations of (7) and (8) such that ${\dot{\theta }}^* = {\hat{\omega }}_{pc}\tilde{\theta }^* + {\dot{\theta }}_{ref}$. The proposed stabilizing solution (9) results in the controlled position dynamics:

10 $$\begin{aligned} \dot{\tilde{\theta }} = - \lambda _{pc}\tilde{\theta } + \tilde{\omega },~\forall t \ge 0, \end{aligned}$$

through the substitution of (9) to the open-loop error dynamics $\dot{\tilde{\theta }} = - \omega _{ref} + \tilde{\omega } + {\dot{\theta }}^*$, which is used in “Analysis” section to analyze the whole system stability and convergence properties considering all control dynamics in “Outer loop” and “Inner loop speed control” sections.

### Inner loop speed control

This section presents the stabilizing solution for the second-order speed error dynamics given by

11 $$\begin{aligned} c_{\omega ,0} \ddot{\tilde{\omega }} = - v_q + d_\omega ,~\forall t \ge 0, \end{aligned}$$

with the coefficient $c_{\omega ,0} \,{:=}\, \frac{J_0 L_0}{K_{m,0}}$ (known) and lumped disturbance $d_\omega \,{:=}\, R_0 i_q + (L_0 N_r i_d + K_{m,0})\omega - {\bar{d}}_q + \frac{B_0 L_0}{K_{m,0}}{\dot{\omega }} - \frac{L_0}{K_{m,0}}\dot{{\bar{d}}}_\omega + \frac{J_0 L_0}{K_{m,0}}\ddot{\omega }_{ref}$, which is obtained using the open-loop speed and current dynamics (4)–(5). The stabilization of the open-loop dynamics (11) requires angular acceleration error (${\tilde{a}} \,{:=}\, {\dot{\omega }}_{ref} - {\dot{\omega }}$) feedback; however, this is not available online without the direct differentiation associated with high-frequency noise magnification. Therefore, an angular acceleration error observer is proposed without requiring any plant true parameter values.

#### Angular acceleration error observer

It follows from the definition ${\tilde{a}} \,{:=}\, {\dot{\omega }}_{ref} - {\dot{\omega }}$ that $\dot{\tilde{\omega }} = {\tilde{a}}$, $\forall t \ge 0$, where the uncertain acceleration error ${\tilde{a}}$ is decomposed as its DC (${\tilde{a}}_0$) and AC ($\Delta {\tilde{a}}$) components such that ${\tilde{a}} = {\tilde{a}}_0 + \Delta {\tilde{a}}$. This representation yields the open-loop system in the chain form, independent from the machine model (2)–(3):

12 $$\begin{aligned} {\dot{\tilde{\omega }}} ={{\tilde{a}},} \end{aligned}$$

13 $$\begin{aligned} {\dot{{\tilde{a}}}}={w,~\forall t \ge 0,} \end{aligned}$$

where $w \,{:=}\, \Delta \dot{{\tilde{a}}}$ and $|w| \le w_{max}$, $\forall t \ge 0$, which corresponds to the genuine idea of this work to solve the model dependence problem in the observer design task. Defining the observer errors $e_{\tilde{\omega }} \,{:=}\, \tilde{\omega } - \tilde{\omega }_{obs}$ and $e_{{\tilde{a}}} \,{:=}\, {\tilde{a}} - {\tilde{a}}_{obs}$ with their estimates $\tilde{\omega }_{obs}$ and ${\tilde{a}}_{obs}$, an acceleration error observer is proposed as

14 $$\begin{aligned} \dot{\tilde{\omega }}_{obs}= (l_{obs,d} + l_{obs,c}) e_{\tilde{\omega }} + {\tilde{a}}_{obs}, \end{aligned}$$

15 $$\begin{aligned} \dot{{\tilde{a}}}_{obs}= l_{obs,d} l_{obs,c} e_{\tilde{\omega }},~\forall t \ge 0, \end{aligned}$$

with observer gains $l_{obs,d} > 0$ (for disturbance attenuation level) and $l_{obs,c} > 0$ (for estimation error convergence rate), whose pole-zero cancellation property results in the exponential convergence property $\lim _{t\rightarrow \infty }{\tilde{a}}_{obs} = {\tilde{a}}$ according to the first-order dynamics ${\dot{e}}_{{\tilde{a}}} = - l_{obs,c} e_{{\tilde{a}}}$ with a sufficient large $l_{obs,d} > 0$. See “Analysis” section for details specifying the admissible range for $l_{obs,d}$.

#### Control law

A proposed stabilizing solution for the second-order open-loop dynamics of (11) is given by

16 $$\begin{aligned} v_q = k_d {\tilde{a}}_{obs} + c_{\omega ,0}\lambda _{sc}{\tilde{a}}_{obs} + k_d \lambda _{sc}\tilde{\omega } + {\hat{d}}_\omega ,~\forall t \ge 0, \end{aligned}$$

with the two design factors $k_d > 0$ and $\lambda _{sc} > 0$. The disturbance estimate ${\hat{d}}_\omega$ comes from the observer-based DOB:

17 $$\begin{aligned} {\dot{\sigma }} = - l \sigma - l^2 c_{\omega ,0} {\tilde{a}}_{obs} + l v_q,~{\hat{d}}_\omega = \sigma + l c_{\omega ,0} {\tilde{a}}_{obs}, \end{aligned}$$

**Figure 1:**
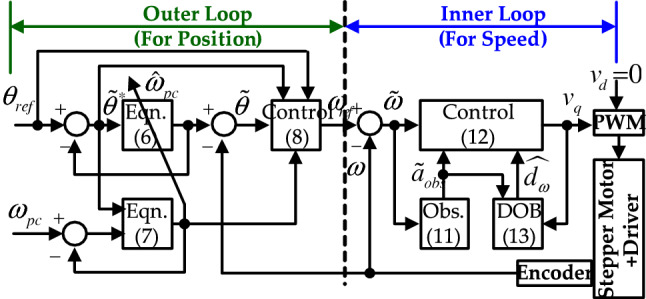
Controller structure.

$\forall t \ge 0$, with design factor $l > 0$. Figure 1 shows the controller structure.

## Analysis

### Subsystem properties

Lemma 1 proves that the time-varying subsystem (7) is stable by augmenting the convergence rate boosting system (8).

#### Lemma 1

The convergence rate booster (7) and (8) guarantees the stability and exponential convergence $\lim _{t\rightarrow \infty }\theta ^* = \theta _{ref}$.

#### Proof

The equivalent form of (7) given by

$$\begin{aligned} \dot{\tilde{\theta }}^* = - \omega _{pc}\tilde{\theta }^* + \tilde{\omega }_{pc}\tilde{\theta }^* \end{aligned}$$

and the update rule (8) turn the Lyapunov function candidate

$$\begin{aligned} V_{m} \,{:=}\, \frac{1}{2}{} {\textbf {x}}_m^T {\textbf {P}}_m {\textbf {x}}_m,~\forall t \ge 0, \end{aligned}$$

with $${\textbf {x}}_m \,{:=}\, \left[ \begin{array}{cc} \tilde{\theta }^*&\tilde{\omega }_{pc} \end{array}\right] ^T$$ and ${\textbf {P}}_m \,{:=}\, \mathrm{diag}\{1, \frac{1}{\gamma _{pc}}\}$ ($\gamma _{pc} > 0$ for the update rule (8)) into

$$\begin{aligned} {\dot{V}}_m &= \tilde{\theta }^*(- \omega _{pc}\tilde{\theta }^* + \tilde{\omega }_{pc}\tilde{\theta }^*) - \tilde{\omega }_{pc} ( (\tilde{\theta }^*)^2 + \rho _{pc}\tilde{\omega }_{pc} ) \\ &= - {\textbf {x}}_m^T {\textbf {Q}}_m {\textbf {x}}_m \\\le & {} -\alpha _m V_m < 0,~\forall t \ge 0, \end{aligned}$$

with ${\textbf {Q}}_m \,{:=}\, \mathrm{diag}\{ \omega _{pc}, \rho _{pc} \}$ (${\hat{\omega }}_{pc}(0) = \omega _{pc} > 0$ and $\gamma _{pc} > 0$ for the update rule (8)) and $\alpha _m \,{:=}\, \frac{2\lambda _{min}({\textbf {Q}}_m)}{\lambda _{max}({\textbf {P}}_m)}$ ($\lambda _{min}((\cdot ))$ and $\lambda _{max}((\cdot ))$ representing the minimum and maximum eigenvalues of the square matrix $(\cdot )$, respectively), which completes the proof. $\square$

Lemma 2 shows that the subsystem comprising (7) and (8) increases the convergence ratio ${\hat{\omega }}_{pc}$ from its initial value ${\hat{\omega }}_{pc}(0) = \omega _{pc} (>0)$, which provides the rationale for a better tracking behavior than the original tracking system (6).

#### Lemma 2

The convergence rate booster (7) and (8) achieves the lower bound on its initial value $\omega _{pc}$. i.e.,

$$\begin{aligned} {\hat{\omega }}_{pc} \ge \omega _{pc},~\forall t \ge 0. \end{aligned}$$

#### Proof

Consider the another form of (8) given by

$$\begin{aligned} \dot{{\hat{\omega }}}_{pc} = - \gamma _{pc} \rho _{pc} {\hat{\omega }}_{pc} + \gamma _{pc} \rho _{pc} \omega _{pc} + \gamma _{pc}(\tilde{\theta }^*)^2,~\forall t \ge 0, \end{aligned}$$

whose solution obtained by the both side integration above has a lower bound as

$$\begin{aligned} {\hat{\omega }}_{pc} &= e^{-\gamma _{pc} \rho _{pc} t} \omega _{pc} + \int _0^t e^{-\gamma _{pc} \rho _{pc} (t-\tau )} (\gamma _{pc} \rho _{pc} \omega _{pc} + \gamma _{pc} (\tilde{\theta }^*)^2 ) d\tau \\ &\ge \omega _{pc},~\forall t \ge 0, \end{aligned}$$

which completes the proof. $\square$

Lemma 3 analyzes the observer error behavior used for the closed-loop convergence analysis.

#### Lemma 3

The acceleration observer driven by (14) and (15) guarantees the exponential convergence

$$\begin{aligned} \lim _{t\rightarrow \infty }e_{\tilde{\omega }} = e^*_{\tilde{\omega }} \end{aligned}$$

where the target trajectory $e^*_{\tilde{\omega }}$ satisfies

18 $$\begin{aligned} {\dot{e}}^*_{\tilde{\omega }} = -l_{obs,c} e^*_{\tilde{\omega }},~\forall t \ge 0, \end{aligned}$$

for a given $l_{obs,c} > 0$ and a sufficient large $l_{obs,d} > 0$ such that $\frac{2w_{max}}{l_{obs,d}}\approx 0$.

#### Proof

Consider the vector form of observer errors defined as $${\textbf {e}}_{obs} \,{:=}\, \left[ \begin{array}{cc} e_{\tilde{\omega }}&e_{{\tilde{a}}}\end{array}\right] ^T$$, which results in the state-space representation for the observer error dynamics using the open-loop system (12)–(13) and the proposed observer (14)–(15) as

$$\begin{aligned} \dot{{\textbf {e}}}_{obs} = {\textbf {A}}_{obs} {\textbf {e}}_{obs} + {\textbf {b}}_1 r + {\textbf {b}}_2 w,~\forall t \ge 0, \end{aligned}$$

where $r \,{:=}\, 0$, $w = \Delta \dot{{\tilde{a}}}$, $${\textbf {A}}_{obs} \,{:=}\, \left[ \begin{array}{cc} -(l_{obs,d} + l_{obs,c}) &{} 1 \\ - l_{obs,d} l_{obs,c} &{} 0 \end{array}\right]$$, $${\textbf {b}}_1 \,{:=}\, \left[ \begin{array}{cc} l_{obs,c}&l_{obs,d}l_{obs,c} \end{array}\right] ^T$$, and $${\textbf {b}}_2 \,{:=}\, \left[ \begin{array}{cc} 0&1 \end{array}\right] ^T$$. This state-space representation and the definition of the observer output with respect to $${\textbf {c}}_{obs} \,{:=}\, \left[ \begin{array}{cc} 1&0 \end{array}\right]$$ as $y_{obs} \,{:=}\, {\textbf {c}}_{obs} {\textbf {e}}_{obs} (=e_{\tilde{\omega }})$, $\forall t \ge 0$, yields that

$$\begin{aligned} {\textbf {c}}_{obs}(s{\textbf {I}} - {\textbf {A}}_{obs})^{-1}{} {\textbf {b}}_1 = \frac{l_{obs,c}(s + l_{obs,d})}{(s+l_{obs,c})(s + l_{obs,d})} = \frac{l_{obs,c}}{s + l_{obs,c}} \end{aligned}$$

and

$$\begin{aligned} {\textbf {c}}_{obs}(s{\textbf {I}} - {\textbf {A}}_{obs})^{-1}{} {\textbf {b}}_2 = \frac{1}{(s+l_{obs,d})(s+l_{obs,c})},~\forall s \in {\mathbb {C}}, \end{aligned}$$

where the proposed observer gain structure causes the pole-zero cancellation in the first calculation result above. These two calculation results give the Laplace transformed observer error output $y_{obs}$ as

$$\begin{aligned} Y_{obs}(s) & = {\textbf {c}}_{obs}(s{\textbf {I}} - {\textbf {A}}_{obs})^{-1}{} {\textbf {b}}_1 R(s) + {\textbf {c}}_{obs} (s{\textbf {I}}-{\textbf {A}}_{obs})^{-1}{} {\textbf {b}}_2 W(s) \\ & = \frac{l_{obs,c}}{s + l_{obs,c}} R(s) + \frac{1}{s + l_{obs,c}}W_F(s),~\forall s \in {\mathbb {C}} \end{aligned}$$

with $Y_{obs}(s)$, *R*(*s*), and *W*(*s*) being the Laplace transforms of $y_{obs}$, $r (=0)$, and *w*, respectively, and $W_F(s) \,{:=}\, \frac{1}{s + l_{obs,d}}W(s)$. The inverse Laplace transform obtains the time domain expression as

$$\begin{aligned} {\dot{e}}_{\tilde{\omega }} = - l_{obs,c} e_{\tilde{\omega }} + w_F,~{\dot{w}}_F = - l_{obs,d}w_F + w,~\forall t \ge 0, \end{aligned}$$

with $w_{F}$ denoting the inverse Laplace transform of $W_F(s)$, which yields the performance error for $\varepsilon _{\tilde{\omega }} \,{:=}\, e^*_{\tilde{\omega }} - e_{\tilde{\omega }}$ as (using (18))

19 $$\begin{aligned} {\dot{\varepsilon }}_{\tilde{\omega }} = - l_{obs,c} \varepsilon _{\tilde{\omega }} - w_F,~{\dot{w}}_F = - l_{obs,d}w_F + w,~\forall t \ge 0. \end{aligned}$$

Define the Lyapunov function candidate as

$$\begin{aligned} V_\varepsilon \,{:=}\, \frac{1}{2}\varepsilon ^2_{\tilde{\omega }} + \frac{\kappa _{w_F}}{2} w_F^2,~\kappa _{w_F} > 0, \end{aligned}$$

which gives along the performance error dynamics (19) using Young’s inequality (e.g., $xy \le \frac{\varepsilon }{2}x^2 + \frac{1}{2\varepsilon }y^2$, $\forall \varepsilon > 0$) as

$$\begin{aligned} {\dot{V}}_\varepsilon & = \varepsilon _{\tilde{\omega }} ( - l_{obs,c}\varepsilon _{\tilde{\omega }} - w_F ) - \frac{\kappa _{w_F}l_{obs,d}}{2}w_F^2 - \kappa _{w_F}\left(\frac{l_{obs,d}}{2}w^2_F - w w_F \right)\\\le & {} - \frac{l_{obs,c}}{2} \varepsilon ^2_{\tilde{\omega }} - \left(\frac{\kappa _{w_F}l_{obs,d}}{2} - \frac{1}{2l_{obs,c}}\right)w_F^2,~\forall t \ge 0,~\forall |w_F| \ge \frac{2w_{max}}{l_{obs,d}}, \end{aligned}$$

with a positive constant $w_{max}$ satisfying $|w| \le w_{max}$, $\forall t \ge 0$. Then, the selection of $\kappa _{w_F} \,{:=}\, \frac{1}{l_{obs,d}} (\frac{1}{l_{obs,c}} + 1)$ removes the indefinite term in the upper bound as

$$\begin{aligned} {\dot{V}}_\varepsilon &\le - \frac{l_{obs,c}}{2}\varepsilon ^2_{\tilde{\omega }} - \frac{1}{2} w_F^2 \\ & \le - \alpha _\varepsilon V_\varepsilon < 0,~\forall t \ge 0, \end{aligned}$$

where $\alpha _e \,{:=}\, \min \{ l_{obs,c}, \frac{1}{\kappa _{w_F}} \}$ subject to a large gain setting $l_{obs,d}$ satisfying $\frac{2w_{max}}{l_{obs,d}}\approx 0$, which completes the proof by the comparison principle in^43^. $\square$

#### Remark 1

The inequality ${\dot{V}}_\varepsilon < 0$ reveals that

20 $$\begin{aligned} {\dot{e}}_{\tilde{\omega }} = -l_{obs,c}e_{\tilde{\omega }},~\forall t \ge 0, \end{aligned}$$

for some setting $l_{obs,d} > 0$. This leads to the reasoning process using the subsystem dynamics of (15) given by

$$\begin{aligned} \ddot{e}_{\tilde{\omega }} = - l_{obs,c} {\dot{e}}_{\tilde{\omega }}\Leftrightarrow & {} \,(\dot{{\tilde{a}}} - \ddot{\tilde{\omega }}_{obs}) = - l_{obs,c} ( {\tilde{a}} - \dot{\tilde{\omega }}_{obs} ) \\\Leftrightarrow & {} \,(\dot{{\tilde{a}}} - ((l_{obs,d} + l_{obs,c}) {\dot{e}}_{\tilde{\omega }} + \dot{{\tilde{a}}}_{obs})) = - l_{obs,c}( {\tilde{a}} - ((l_{obs,d} + l_{obs,c})e_{\tilde{\omega }} + {\tilde{a}}_{obs}) ) \end{aligned}$$

which concludes that

21 $$\begin{aligned} {\dot{e}}_{{\tilde{a}}} = -l_{obs,c} e_{{\tilde{a}}},~\forall t \ge 0, \end{aligned}$$

verifying the exponential acceleration error estimation convergence used for the remaining analysis.

Lemma 4 analyzes the disturbance estimate behavior from DOB used for the closed-loop convergence analysis using the result of Lemma 3.

#### Lemma 4

The DOB (17) ensures that

22 $$\begin{aligned} \dot{{\tilde{d}}}_\omega = - l {\tilde{d}}_\omega - l \gamma e_{{\tilde{a}}} + {\dot{d}}_\omega ,~\forall t \ge 0, \end{aligned}$$

for some $\gamma > 0$, where ${\tilde{d}}_\omega \,{:=}\, d_\omega - {\hat{d}}_\omega$, $\forall t \ge 0$.

#### Proof

It follows from the output of DOB in the right side of (17) that

$$\begin{aligned} \dot{{\hat{d}}}_\omega & = {\dot{\sigma }} + l c_{\omega ,0}\dot{{\tilde{a}}}_{obs} = - l( {\hat{d}}_\omega - lc_{\omega ,0}{\tilde{a}}_{obs} ) - l^2 c_{\omega ,0} {\tilde{a}}_{obs} + l v_q + l c_{\omega ,0} \ddot{\tilde{\omega }} - l c_{\omega ,0} {\dot{e}}_{{\tilde{a}}} \\ & = l ( c_{\omega ,0}\ddot{\tilde{\omega }} + v_q - {\hat{d}}_\omega ) - l c_{\omega ,0} {\dot{e}}_{{\tilde{a}}} \\ & = l ( d_\omega - {\hat{d}}_\omega ) + l l_{{\tilde{a}}}c_{\omega ,0} e_{{\tilde{a}}},~\forall t \ge 0, \end{aligned}$$

where the DOB (17) yields the second equation and the last equation is obtained from the relationships (11) and (21), which completes the proof. $\square$

### Closed-loop stability and convergence

Interestingly, the proposed controller results in the order reduction of the closed-loop speed dynamics by the stable pole-zero cancellation, which is proven in Lemma 5.

#### Lemma 5

The proposed voltage updating law of (16) allows the speed error to be satisfied:

23 $$\begin{aligned} \dot{\tilde{\omega }} = - \lambda _{sc}\tilde{\omega } + {\tilde{d}}_{\omega ,F} + e_{{\tilde{a}},F}, \end{aligned}$$

with the perturbations ${\tilde{d}}_{\omega ,F}$ and $e_{{\tilde{a}},F}$ such that

$$\begin{aligned} \dot{{\tilde{d}}}_{\omega ,F} = - b_1 {\tilde{d}}_{\omega ,F} + b_2 {\tilde{d}}_\omega ~\text{ and }~{\dot{e}}_{{\tilde{a}},F} = - b_1 e_{{\tilde{a}},F} + b_3 e_{{\tilde{a}}},~\forall t \ge 0, \end{aligned}$$

for some $$b_i > 0$$, $$i=1,2,3$$.

#### Proof

The controlled speed error system is obtained as

$$\begin{aligned} c_{\omega ,0}\ddot{\tilde{\omega }} & = - k_d {\tilde{a}}_{obs} - c_{\omega ,0} \lambda _{sc}{\tilde{a}}_{obs} - k_d \lambda _{sc}\tilde{\omega } + {\tilde{d}}_\omega \\ & = - k_d \dot{\tilde{\omega }} + c_{\omega ,0}\lambda _{sc}(\dot{\tilde{\omega }}_{ref} - \dot{\tilde{\omega }}) + k_d \lambda _{sc} (\tilde{\omega }_{ref} - \tilde{\omega }) + {\tilde{d}}_\omega + (k_d + c_{\omega ,0}\lambda _{sc}) e_{{\tilde{a}}},~\forall t \ge 0, \end{aligned}$$

with the combination of (11) and (16) and $\tilde{\omega }_{ref} \,{:=}\, 0$. Taking the Laplace transform, it holds that

$$\begin{aligned} (c_{\omega ,0}s^2 + (k_d + c_{\omega ,0}\lambda _{sc})s + k_d\lambda _{sc} ) \tilde{\Omega } (s) = \lambda _{sc} ( c_{\omega ,0}s + k_d )\tilde{\Omega }_{ref}(s) + {\tilde{D}}_\omega (s) + (k_d + c_{\omega ,0}\lambda _{sc})E_{{\tilde{a}}}(s) \end{aligned}$$

which yields that

$$\begin{aligned} (s + \lambda _{sc})\tilde{\Omega }(s) = \lambda _{sc}\tilde{\Omega }_{ref} (s) + {\tilde{D}}_{\omega ,F}(s) + E_{{\tilde{a}},F}(s) \end{aligned}$$

where the pole-zero cancellation from the factorization

$$\begin{aligned} (c_{\omega ,0}s^2 + (k_d + c_{\omega ,0}\lambda _{sc})s + k_d\lambda _{sc} ) = (c_{\omega ,0}s + k_d)(s + \lambda _{pc}) \end{aligned}$$

is applied and ${\tilde{D}}_{\omega ,F}(s) = \frac{\frac{1}{c_{\omega ,0}}}{s + \frac{k_d}{c_{\omega ,0}}}{\tilde{D}}_\omega (s)$ and $E_{{\tilde{a}},F}(s) = \frac{\frac{k_d + c_{\omega ,0}\lambda _{sc}}{c_{\omega ,0}}}{s + \frac{k_d }{c_{\omega ,0}}} E_{{\tilde{a}}}(s)$, which completes the proof. $\square$

Finally, Theorem 1 asserts the main result.

#### Theorem 1

The proposed controller comprising (14)–(17) ensures the exponential convergence (control objective)

$$\begin{aligned} \lim _{t\rightarrow \infty }\theta = \theta ^* \end{aligned}$$

for $l > 0$ such that $\frac{2d_{max}}{l}\approx 0$, where $|{\dot{d}}_\omega | \le d_{max}$, $\forall t \ge 0$.

#### Proof

The vector $${\textbf {x}} \,{:=}\, \left[ \begin{array}{cccc} \tilde{\theta }&\tilde{\omega }&{\tilde{d}}_{\omega ,F}&e_{{\tilde{a}},F} \end{array}\right] ^T$$ leads to the augmented system given by

$$\begin{aligned} \dot{{\textbf {x}}} = {\textbf {A}}_x {\textbf {x}} + {\textbf {b}}_{x,d} {\tilde{d}}_\omega + {\textbf {b}}_{x,e}e_{{\tilde{a}}} \end{aligned}$$

where $${\textbf {A}}_x \,{:=}\, \left[ \begin{array}{cccc} -\lambda _{pc} &{} 1 &{} 0 &{} 0 \\ 0 &{} -\lambda _{sc} &{} 1 &{} 1 \\ 0 &{} 0 &{} -b_1 &{} 0 \\ 0 &{} 0 &{} 0 &{} -b_1 \end{array}\right]$$, $${\textbf {b}}_{x,d} \,{:=}\, \left[ \begin{array}{cccc} 0 \\ 0 \\ b_2 \\ 0 \end{array}\right]$$, and $${\textbf {b}}_{x,e} \,{:=}\, \left[ \begin{array}{cccc} 0 \\ 0 \\ 0 \\ b_3 \end{array}\right]$$. The stability of ${\textbf {A}}_x$ picks an unique solution ${\textbf {P}}_x > {\textbf {0}}$ such that ${\textbf {A}}_x^T {\textbf {P}}_x + {\textbf {P}}_x {\textbf {A}}_x = - {\textbf {I}}$, which defines the Lyapunov function candidate defined as

$$\begin{aligned} V \,{:=}\, \frac{1}{2}{} {\textbf {x}}^T {\textbf {P}}_x {\textbf {x}} + \frac{\kappa _{d}}{2} {\tilde{d}}^2_\omega + \frac{\kappa _a}{2}e^2_{{\tilde{a}}},~\kappa _d> 0,~\kappa _a > 0,~\forall t \ge 0. \end{aligned}$$

The above augmented system and (22) gives its time derivative along the trajectories as

$$\begin{aligned} {\dot{V}} = {\textbf {x}}^T {\textbf {P}}_x ({\textbf {A}}_x {\textbf {x}} + {\textbf {b}}_{x,d} {\tilde{d}}_\omega + {\textbf {b}}_{x,e}e_{{\tilde{a}}}) + \kappa _d {\tilde{d}}_\omega ( -\frac{l}{2} {\tilde{d}}_\omega - l\gamma e_{{\tilde{a}}} ) - \kappa _a l_{obs,c}e^2_{{\tilde{a}}} -\kappa _d ( \frac{l}{2}{\tilde{d}}^2_\omega - {\tilde{d}}_\omega {\dot{d}}_\omega ) \end{aligned}$$

with its upper bound by Young’s inequality (e.g., ${\textbf {p}}^T {\textbf {q}} \le \frac{\varepsilon }{2}\Vert {\textbf {p}}\Vert ^2 + \frac{1}{2\varepsilon }\Vert {\textbf {q}}\Vert ^2$, $\forall \varepsilon > 0$):

$$\begin{aligned} {\dot{V}} \le - \frac{1}{3}\Vert {\textbf {x}}\Vert ^2 - (\frac{\kappa _d l}{2} -\frac{\Vert {\textbf {P}}_x\Vert ^2 b^2_2}{4} - \frac{1}{2}){\tilde{d}}^2_\omega - (\kappa _a l_{obs,c} - \frac{\Vert {\textbf {P}}_x\Vert ^2 b_3^2}{4} - \frac{\kappa _d^2 l^2 \gamma ^2}{2} )e^2_{{\tilde{a}}},~\forall t \ge 0,~\forall |{\tilde{d}}_\omega | \ge \frac{2d_{max}}{l}. \end{aligned}$$

Then, the selections of $\kappa _d \,{:=}\, \frac{2}{l} ( \frac{\Vert {\textbf {P}}_x\Vert ^2 b^2_2}{4} + 1 )$ and $\kappa _a \,{:=}\, \frac{1}{l_{obs,c}}(\frac{\Vert {\textbf {P}}_x\Vert ^2 b_3^2}{4} + \frac{\kappa _d^2 l^2 \gamma ^2}{2} + \frac{1}{2})$ rearrange the upper bound of ${\dot{V}}$ such that

$$\begin{aligned} {\dot{V}}\le & {} - \frac{1}{3}\Vert {\textbf {x}}\Vert ^2 - \frac{1}{2}{\tilde{d}}^2_\omega - \frac{1}{2}e^2_{{\tilde{a}}} \\\le & {} - \alpha V,~\forall t \ge 0, ~\forall |{\tilde{d}}_\omega | \ge \frac{2d_{max}}{l}, \end{aligned}$$

where $\alpha \,{:=}\, \min \{ \frac{2}{3\lambda _{min}({\textbf {P}}_x)}, \frac{1}{\kappa _d}, \frac{1}{\kappa _a} \}$, completing the proof. $\square$

#### Remark 2

Based on the above analysis results, this remark finalizes this section by suggesting a tuning process of the proposed controller comprising the speed (inner) and position (outer) loops shown in Fig. 1 as follows:

1. (speed loop for steps 1-4) Using well-working speed controller, e.g., PI controller with a constant speed reference $\omega _{ref}$, tune the observer gains $l_{obs,c}$ and $l_{obc,d}$ for a rapid estimation error convergence in accordance with Remark 1; for example, first, choose $l_{obs,c}$ such that $l_{obs,c} \ge 50$ for ${\dot{e}}_{\tilde{\omega }} = -l_{obs,c}e_{\tilde{\omega }}$ and, second, increase $l_{obs,d}$ holding $l_{obs,d}\gg l_{obs,c}$.
2. Tune the DOB gain $l > 0$ to assign the cut-off frequency ($l = 2\pi f_l$ rad/s, equivalently, $f_{l} = \frac{l}{2\pi }$ Hz) for the transfer function $\frac{{\hat{D}}_\omega (s)}{D_{\omega }(s)} = \frac{l}{s + l}$ (obtained from (22) under the condition $e_{{\tilde{a}}} = 0$); for example, choose $f_{l} \ge 2$ Hz such that $l \ge 2\pi f_l = 12.6$ rad/s.
3. Using the proposed speed controller (16) with a constant speed reference $\omega _{ref}$ (for step 3 and 4), select $f_{sc} \in [10, 30]$ yielding $\lambda _{sc} \in [2\pi 10 (=\lambda _{sc,min}), 2\pi 30 (=\lambda _{sc,max})]$ (e.g., $\lambda _{sc} = 2\pi f_{sc}$ rad/s and $f_{sc} = \frac{\lambda _{sc}}{2\pi }$ Hz); the maximum interval value may be increased depending on the hardware specification.
4. Increase the active damping coefficient $k_d$ (for example, $k_d \ge 0.001$) for an acceptable speed tracking response ${\dot{\omega }} \approx \lambda _{sc} \tilde{\omega }$ (some iteration between step 3 and 4 may be required).
5. (position loop for steps 5-7) Using the proposed position controller (9) with a constant position reference $\theta _{ref}$, set $\gamma _{pc} = \rho _{pc} = 0$ and select $f_{pc} \in [0.1, 5]$ yielding $[2\pi 0.1 (=\omega _{pc,min}), 2\pi 5 (=\omega _{pc,max})]$; the maximum interval value may depend on the hardware specification.
6. Increase $\lambda _{pc}$ (for example, $\lambda _{pc} \ge 10$) until an acceptable position tracking response ${\dot{\theta }} \approx \omega _{pc}\tilde{\theta }$ is obtained (some iteration between step 5 and 6 may be required).
7. Increase $\gamma _{pc}$ and $\rho _{pc} = \frac{\kappa _{pc}}{\gamma _{pc}}$ with $\kappa _{pc} > 0$ until the peak value and restoration rate of the convergence rate are acceptable; for example, choose $\gamma _{pc} \ge 1$ and $\kappa _{pc} \ge \frac{\gamma _{pc}}{2}$.

This process results in the controller tuning values used in “Experimental results” section.

## Experimental results

**Figure 2:**
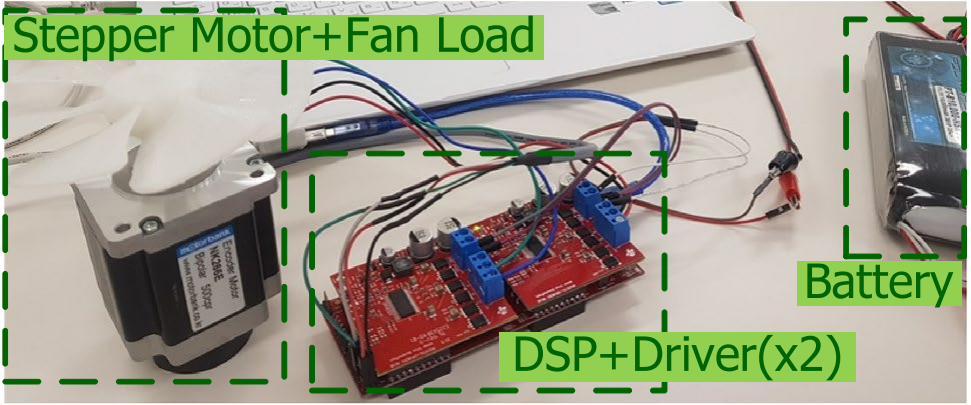
Experimental setup.

This section experimentally demonstrates the position-tracking performance improvements by comparison with an extant controller. A 10-W stepping motor embedding an encoder for position feedback (model:NK266E-02AT) and Texas Instrument (TI) LAUNCHXL-F28069M (digital signal processor) were used for experimental setup shown in Fig. 2 (see^41^ for more detailed configuration).

The controller tuning results are summarized as (convergence rate booster) $f_{pc} = 0.2$ Hz such that ${\hat{\omega }}_{pc}(0) = \omega _{pc} = 2\pi 0.2$ rad/s, $\gamma _{pc} = 2$, $\rho _{pc} = 0.5/\gamma _{pc}$, (control gain) $\lambda _{pc} = 1.25$, $\lambda _{sc} = 125.6$, $k_d = 0.1$, (observer gain) $l_{obs,c} = 100$, $l_{obs,d} = 500$, and (DOB gain) $l = 20$. This study chooses the FL controllers (in^30^) for the comparison study, including the active damping and feed-forward term, given by: $v_x = K_{P,cc} {\tilde{i}}_x + K_{I,cc} \int _0^t {\tilde{i}}_x d\tau - p_{x,0}$, $i_{q,ref} = \frac{1}{K_{m,0}} ( - k_d \omega + K_{P,sc}\tilde{\omega } + K_{I,sc} \int _0^t \tilde{\omega }d\tau ),~(i_{d,ref} = 0)$, $\omega _{ref} = k_{pc}\tilde{\theta }$, $\forall t \ge 0$, $x=d,q$, with feedback gains $K_{P,cc} = L_0 \lambda _{cc}$, $K_{I,cc} = R_0 \lambda _{cc}$, $K_{P,sc} = J_0 \lambda _{sc}$, $K_{I,sc} = (k_d + B_0) \lambda _{sc}$, and carefully tuned value $k_d = 0.01$. The current cut-off frequency was tuned to $\lambda _{cc} = 314$ by applying the same settings for the position and speed loops in the proposed controller.

### Tracking task

**Figure 3:**
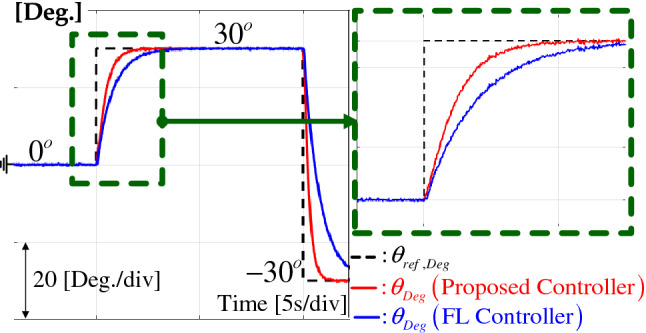
Position response comparison from tracking task.

**Figure 4:**
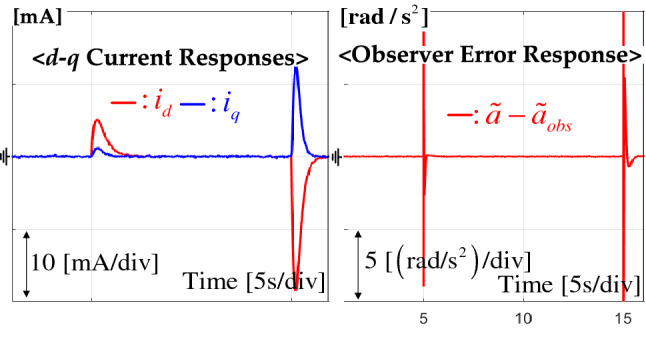
*d*–*q* axis current and angular acceleration estimation error responses from tracking task.

**Figure 5:**
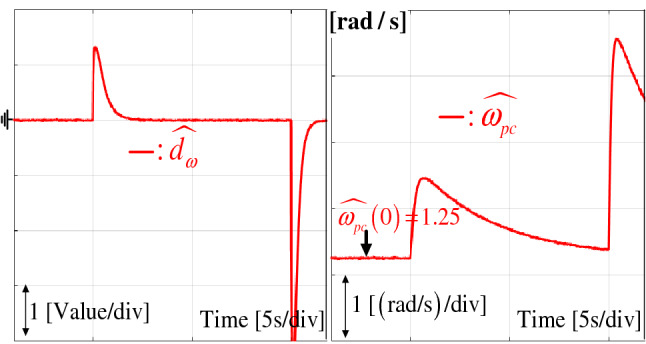
DOB and convergence rate responses from tracking task.

For the stair position reference, Fig. 3 demonstrates an improved tracking performance from the convergence rate boosting mechanism and performance recovery property proved in Theorem 1. Figure 4 presents the *d*-*q* axis current and observer error responses. Figure 5 shows the DOB and convergence rate booster responses. The intended convergence rate behavior improves the tracking performance as shown in Fig. 3.

### Frequency response

**Figure 6:**
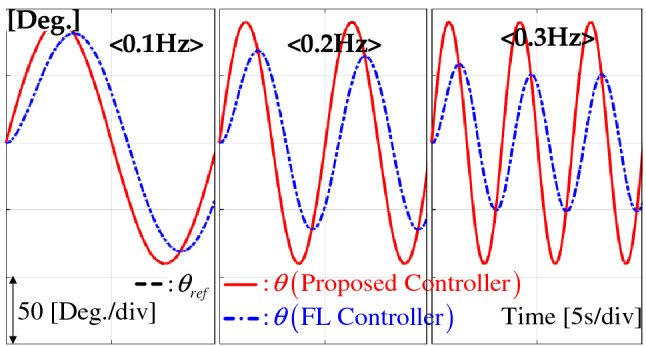
Position responses for sinusoidal references of 0.1, 0.2, and 0.3 Hz.

The proposed controller robustly forces the position motion to be the first-order tracking error system (7) by the beneficial capability shown in Theorem 1. This section verifies this advantage for the sinusoidal reference signals 0.1, 0.2, and 0.3 Hz. Figure 6 shows that the proposed controller provides the desired position-tracking behavior without any magnitude and phase distortion unlike the FL controller.

### Regulation task

**Figure 7:**
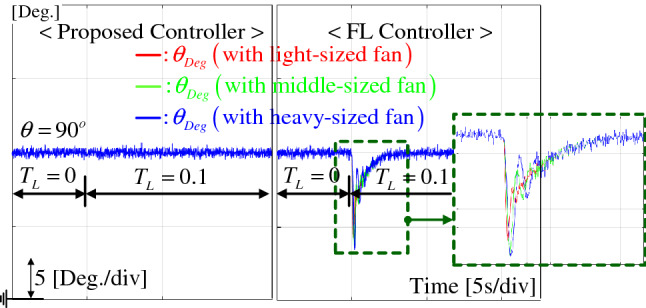
Position response comparison under regulation task for light-, medium-, and heavy-sized fan loads.

**Figure 8:**
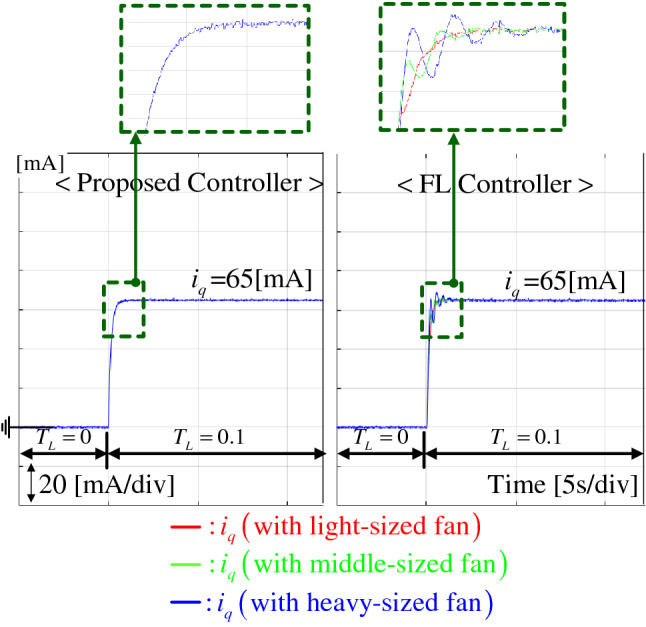
*q*-axis current response comparison under regulation task for light-, medium-, and heavy-sized fan loads.

To evaluate the regulation performance, a load torque of $T_L = 0.1$ Nm was abruptly applied to the closed-loop system (by suddenly attaching the rotating wheel to the rotor) operating at $\theta _{ref,Deg} = 90^{\circ }$ under the three load conditions, such as light-, medium-, and heavy-sized fan. Figure 7 presents that the proposed technique accomplishes drastic regulation performance improvement under different loads compared with the FL controller that provides the magnified undershoots with oscillations and performance inconsistency for different load conditions. The corresponding *q*-axis current responses are compared in Fig. 8, which exhibits the improved current regulation performance by the proposed controller despite in the absence of current feedback.

## Conclusions

The proposed current sensorless feedback system was driven by a PD-type controller incorporating the novel techniques, such as a convergence rate booster, angular acceleration error observer (model-free), and DOB without requiring the true motor parameters. This study has both proved the beneficial closed-loop properties and experimentally confirmed the practical advantages for tracking tasks. However, an acceptable setting for numerous design parameters should be identified through a systematic process, which is will be conducted in a future study.

## Data Availability

The datasets used and/or analysed during the current study available from the corresponding author on reasonable request.
